# NCD Prevention and Control: Sustainable and Comprehensive Solutions; A Response to Recent Commentaries

**DOI:** 10.15171/ijhpm.2019.129

**Published:** 2019-12-08

**Authors:** Viroj Tangcharoensathien, Orana Chandrasiri, Watinee Kunpeuk, Kamolphat Markchang, Nattanicha Pangkariya

**Affiliations:** International Health Policy Program, Ministry of Public Health, Nonthaburi, Thailand.

The four commentaries on an *IJHPM* editorial “Addressing NCDs: Challenges from industry market promotion and interferences,”^1^ further exemplify the tactics used by tobacco, alcohol and unhealthy food industry in interfering governments’ efforts to counteract the commercial determinants of non-communicable disease (NCD); in particular the best buys interventions. These interventions, in particular increase tax and retail price of tobacco and alcohol; control advertising and marketing and limit the availability of these products, clearly challenge and invite industry’s heavy fight back.^2^ The commentaries also raise concerns about the high level of industry interference, focusing on the emerging markets in low- and middle-income countries in Asia and Africa, in particular in the context of poor legislation and weak regulatory environment.^3^ Table provides alarming trends of sugar consumption where excessive consumption contributes to NCD, between 2008 and 2027 in selected countries.

**Table:** Sugar Consumption Trend 2008-2027, Selected Countries

	**Consumption (kg)**		**Growth ( %)** ^c^		**Per Capita (kg)**		**Growth ( %)** ^c^	
	**Average 2015-2017est**	**2027**	**2008-2017**	**2018-2027**	**Average 2015-2017est**	**2027**	**2008-2017**	**2018-2027**
**World**	167 118	197 870	1.66	1.48	22.3	23.7	0.47	0.51
**North America**	11 416	12 871	1.16	1.07	31.8	33.2	0.38	0.35
Canada	1175	1263	-0.09	0.73	32.4	31.8	-1.11	-0.09
United States	10 241	11 608	1.31	1.11	31.8	33.4	0.56	0.40
**Latin America**	26 660	29 906	0.04	1.07	41.8	42.6	-1.09	0.21
Argentina	1658	1983	0.23	1.69	37.8	41.1	-0.80	0.84
Brazil	11 038	11 952	-1.10	0.82	53.2	53.7	-1.99	0.22
Chile	780	879	1.23	1.02	43.6	45.5	0.33	0.35
Colombia	1849	2214	2.44	1.66	38.0	42.2	1.44	1.01
Mexico	4462	4997	0.30	1.03	35.0	34.7	-1.11	-0.04
Paraguay	134	157	1.58	1.38	20.0	20.6	0.24	0.25
**Europe**	27 113	26 830	0.29	-0.15	36.0	35.6	0.19	-0.14
European Union	18 502	17 910	0.60	-0.36	36.4	34.9	0.43	-0.41
Russia	5713	6011	0.18	0.48	39.7	42.4	0.10	0.65
Ukraine	1639	1639	-1.94	-0.21	36.9	39.1	-1.46	0.32
**Africa**	19 191	26 926	3.63	3.07	15.7	16.9	0.99	0.66
Egypt	3508	4739	3.89	2.65	36.7	41.3	1.72	1.02
Ethiopia	491	704	3.75	3.32	4.8	5.4	1.10	1.03
Nigeria	1593	2347	5.32	3.59	8.6	9.6	2.56	1.03
South Africa	1931	2189	1.32	1.09	34.5	34.8	-0.02	0.06
**Asia**	81 254	99 681	2.37	1.75	18.2	20.5	1.31	1.01
China^a^	16 145	20 234	2.38	1.90	11.5	14.0	1.84	1.70
India	24 717	31 124	1.94	1.99	18.7	21.1	0.69	1.01
Indonesia	6622	8343	4.06	1.93	25.4	28.8	2.77	1.02
Iran	2492	2652	0.99	0.51	31.0	30.3	-0.23	-0.23
Japan	2108	2039	-0.46	-0.35	16.5	16.5	-0.37	-0.00
Kazakhstan	498	546	1.44	0.81	27.7	27.4	-0.06	-0.06
Korea	1591	1784	3.06	0.88	31.3	34.0	2.65	0.60
Malaysia	1792	2203	3.81	1.73	57.5	61.7	2.03	0.50
Pakistan	5085	6748	2.69	2.63	26.3	28.9	0.58	0.90
Philippines	2203	2850	2.59	2.39	21.3	23.6	0.94	0.97
Saudi Arabia	1225	1597	3.70	2.31	38.0	41.8	0.92	0.85
Thailand	2965	3142	3.03	0.43	43.1	45.1	2.60	0.35
Turkey	2395	2624	1.56	0.62	30.1	30.2	0.01	0.00
Vietnam	1561	1960	5.03	1.89	16.5	18.8	3.89	1.01
**Oceania**	1483	1656	1.60	1.10	37.8	36.7	0.02	-0.15
Australia	1163	1294	1.73	1.05	48.2	47.2	0.22	-0.08
New Zealand	220	236	0.35	0.70	47.2	46.2	-0.72	-0.11
Developed countries	46 337	48 216	0.61	0.33	32.6	32.8	0.20	0.04
Developing countries	120 781	149 654	2.09	1.88	19.9	21.8	0.70	0.75
Least developed countries	7573	10 928	5.23	3.19	9.5	10.7	2.77	0.91
OECD^b^	43 683	45 850	0.83	0.39	32.8	32.8	0.27	-0.00
BRICS	59 545	71 511	1.24	1.60	19.0	21.4	0.39	1.03

Abbreviations: OECD, Organisation for Economic Co-operation and Development; BRICS, Brazil, Russia, India, China and South Africa. *Note:* Marketing year: See Glossary of Terms for definitions. Average 2015-17est: Data for 2017 are estimated. Sugar data are expressed on a tel quel basis. Disclaimer: http://oe.cd/disclaimer. ^a^ Refers to mainland only. The economies of Chinese Taipei, Hong Kong (China) and Macau (China) are included in the Asia aggregate. ^b^ Excludes Iceland but includes all EU28 member countries. ^c^ Least-squares growth rate (see glossary). Source: OECD (2018), “Table A.23.2 - Sugar projections: Consumption, food,” in *OECD-FAO Agricultural Outlook 2018-2027* , OECD Publishing, Paris, https://doi.org/10.1787/agr_outlook-2018-table140-en.

Not only four tactics identified by the editorial, the use of “trade and investment disputes” through the Technical Barrier to Trade platform of the World Trade Organization is powerful in discouraging government making bold efforts.^4^ For example, Thailand’s efforts of introducing pictorial health warning on alcohol were challenged by industries and World Trade Organization’s members including European Union as an excessive barrier to trade.^5^

The industry commonly uses the discourse that “alcohol related problems are in the person and not in the bottle.”^6^ Unlike other psychotropic substances, alcohol, classified by International Agency for Research on Cancer (IARC) as group 1 carcinogenic to humans, has not yet been controlled by international binding instrument. Despite these gloomy situations; the Scottish government was successful in counteracting legal threat by alcohol industry in introducing minimum unit price of alcohol to prevent promotion of consumption through lowering price.^7^

*“Alcoholic beverages were declared as group 1 ‘carcinogenic to humans’ by the IARC Monographs Programme , first in 1988 and then again in 2007 and in 2010.* ^8^ *Tumour types caused by drinking alcoholic beverages include cancers of the oral cavity, pharynx, larynx, oesophagus , liver, colorectum, and female breast. For renal cell carcinoma and non-Hodgkin lymphoma, there is ‘evidence suggesting lack of carcinogenicity’ for alcohol consumption.”* ^9^

We strongly support the proposal for a legally binding international agreement.^10^ Learning from World Health Organization (WHO) Framework Convention for Tobacco Control, the WHO Member States should negotiate the development of an international agreement on alcohol control to help foster coherent policies and regulatory measures against industry interferences and aggressive market promotion.

Further, all heath conferences, meetings, events and workshops sponsored or organized by WHO, governments and academia should be alcohol free. This sets the social norm and precedence in addressing harmful use of alcohol through civic actions. We call WHO, as world health leader, leading the “alcohol-free role model.”

We strongly support that government officials shall not engage in or associate with tobacco and alcohol industry even under the so-called corporate social responsibility. The Foundation for a Smoke-Free World, launched in 2017, was vowed as an “independent” research funding body but it was fully funded by Philip Morris Inc. The Foundation can support research and development of new smokeless tobacco products.^11^

Capacity of government alone is inadequate to respond to market promotion and sales through internet. This fosters the needs for international instrument and collective efforts across countries. Rampant global and cross-border market promotion through internet sales via application in smart phone by tobacco industries; their website promotes sales through coupons, games, social activities/events and sweepstakes or contests.^12^ Strong national capacity, international collaboration and vigilance by civil society organization can synergistically respond to these emerging challenges.

Evidence shows neither self-regulation nor collaborative initiatives are effective in achieving public health objectives to prevent NCD in particular in the context of aggressive marketing, conflicts of interest between industries and government officials and weak regulatory capacities in most low- and middle-income countries.

At national level, policy coherence and effective multi-sectoral actions to safeguard health of the population are critical. Philippines have demonstrated an effective collaboration between Department of Finance and Health in introducing sugar sweetened beverage tax through legislation.^13^ There is a need for improve governance and leadership which translate into political commitment to support whole-of-government and health-in-all-policies approaches. The commitment should be translated into budget allocation for NCD prevention and control. There are good practices on the use of earmarked tax from tobacco and alcohol for active health promotion.^14^ There is a need to boost the implementation capacity for NCD prevention and control and the application of 16 best-buy interventions.

In addition to multi-sectoral actions; there is an urgent need to mobilize the legal workforce, strengthen legal capacity and support effective use of law at the national level. Legal and regulatory actions are required to be at the centre of national NCD action plans. Strengthening legal capacity requires high-level leadership from global and national leaders, enacting evidence-based legislation and building legal capacities.^15^ National legal capacity can address the challenges from the use of trade and investment agreement by industry.

## Ethical issues

Not applicable.

## Competing interests

Authors declare that they have no competing interests.

## Authors’ contributions

All authors involved in the conceptualize the paper, design, and analysis. VT proposed the structure and main ideas. OC, WK, KM, and NP summarized key points from four commentaries. All authors read and approved the final manuscript.

## References

[1] Tangcharoensathien V, Chandrasiri O, Kunpeuk W, Markchang K, Pangkariya N (2019). Addressing NCDs: challenges from industry market promotion and interferences. Int J Health Policy Manag.

[2] Watt RG, Sheiham A (2012). Integrating the common risk factor approach into a social determinants framework. Community Dent Oral Epidemiol.

[3] Delobelle P (2019). Big tobacco, alcohol, and food and NCDs in LMICs: an inconvenient truth and call to action: Comment on “Addressing NCDs: challenges from industry market promotion and interferences. ” Int J Health Policy Manag.

[4] Cowling K, Magraw D (2019). Addressing NCDs: Protecting health from trade and investment law: Comment on “Addressing NCDs: Challenges from industry market promotion and interferences. ” Int J Health Policy Manag.

[5] Lester S. More on Thai Liquor Labeling. November 28, 2010. https://ielp.worldtradelaw.net/2010/11/more-on-thail-liquor-labeling.html. Accessed November 18, 2019.

[6] O’brien P, Mitchell A (2018). On the Bottle: Health Information, Alcohol Labelling and the WTO Technical Barriers to Trade Agreement. QUT Law Review.

[7] Woodhouse J. Alcohol: minimum pricing. August 29, 2019. https://bit.ly/2D3uESX. Accessed November 18, 2019.

[8] Baan R, Straif K, Grosse Y (2007). Carcinogenicity of alcoholic beverages. Lancet Oncol.

[9] Rehm J, Shield K (2014). Alcohol consumption In: Stewart BW, Wild CP, eds World Cancer Report 2014. Lyon, France: International Agency for Research on Cancer.

[10] Casswell S (2019). Addressing NCDs: penetration of the producers of hazardous products into global health environment requires a strong response: Comment on “Addressing NCDs: challenges from industry market promotion and interferences. ” Int J Health Policy Manag.

[11] Chapman S (2017). Tobacco giant wants to eliminate smoking. BMJ.

[12] O’Brien EK, Navarro MA, Hoffman L (2018). Mobile website characteristics of leading tobacco product brands: cigarettes, smokeless tobacco, e-cigarettes, hookah and cigars. Tob Control.

[13] Onagan FCC, Ho BLC, Chua KKT (2019). Development of a sweetened beverage tax, Philippines. Bull World Health Organ.

[14] Pongutta S, Suphanchaimat R, Patcharanarumol W, Tangcharoensathien V (2019). Lessons from the Thai Health Promotion Foundation. Bull World Health Organ.

[15] Magnusson RS, McGrady B, Gostin L, Patterson D, Abou Taleb H (2019). Legal capacities required for prevention and control of noncommunicable diseases. Bull World Health Organ.

